# A Privacy-Preserving Log-Rank Test for the Kaplan-Meier Estimator With Secure Multiparty Computation: Algorithm Development and Validation

**DOI:** 10.2196/22158

**Published:** 2021-01-18

**Authors:** Marcel von Maltitz, Hendrik Ballhausen, David Kaul, Daniel F Fleischmann, Maximilian Niyazi, Claus Belka, Georg Carle

**Affiliations:** 1 Chair of Network Architectures and Services Department of Informatics Technical University of Munich, TUM Garching Germany; 2 Department of Radiation Oncology University Hospital Ludwig-Maximilians-Universität München, LMU Munich Germany; 3 German Cancer Consortium (DKTK) partner site Munich Munich Germany; 4 Department of Radiation Oncology Charité - University Medicine Berlin Germany; 5 German Cancer Consortium (DKTK) partner site Berlin Berlin Germany; 6 German Cancer Research Center (DKFZ) Heidelberg Germany

**Keywords:** privacy, data protection, privacy preservation, multicentric studies, secure multiparty computation, cryptography

## Abstract

**Background:**

Patient data is considered particularly sensitive personal data. Privacy regulations strictly govern the use of patient data and restrict their exchange. However, medical research can benefit from multicentric studies in which patient data from different institutions are pooled and evaluated together. Thus, the goals of data utilization and data protection are in conflict. Secure multiparty computation (SMPC) solves this conflict because it allows direct computation on distributed proprietary data—held by different data owners—in a secure way without exchanging private data.

**Objective:**

The objective of this work was to provide a proof-of-principle of secure and privacy-preserving multicentric computation by SMPC with real-patient data over the free internet. A privacy-preserving log-rank test for the Kaplan-Meier estimator was implemented and tested in both an experimental setting and a real-world setting between two university hospitals.

**Methods:**

The domain of survival analysis is particularly relevant in clinical research. For the Kaplan-Meier estimator, we provided a secure version of the log-rank test. It was based on the SMPC realization SPDZ and implemented via the FRESCO framework in Java. The complexity of the algorithm was explored both for synthetic data and for real-patient data in a proof-of-principle over the internet between two clinical institutions located in Munich and Berlin, Germany.

**Results:**

We obtained a functional realization of an SMPC-based log-rank evaluation. This implementation was assessed with respect to performance and scaling behavior. We showed that network latency strongly influences execution time of our solution. Furthermore, we identified a lower bound of 2 Mbit/s for the transmission rate that has to be fulfilled for unimpeded communication. In contrast, performance of the participating parties have comparatively low influence on execution speed, since the peer-side processing is parallelized and the computational time only constitutes 30% to 50% even with optimal network settings. In the real-world setting, our computation between three parties over the internet, processing 100 items each, took approximately 20 minutes.

**Conclusions:**

We showed that SMPC is applicable in the medical domain. A secure version of commonly used evaluation methods for clinical studies is possible with current implementations of SMPC. Furthermore, we infer that its application is practically feasible in terms of execution time.

## Introduction

Medical research is in large part based on clinical patient data. In this domain, particularly strict data protection regulations apply, which increase the effort required to utilize these data and leverage their full potential. In fact, it is a common regulatory requirement that patient data must not leave the custody of the hospital. However, great scientific value and substantial patient benefit could be achieved if institutions were permitted to pool their data to reach more accurate and more reliable conclusions across the entirety of their patient populations. In the dawning era of multiomics, eHealth and wearables, individual big data, and nonlinear evaluation such as machine learning, the problem is exponentially exacerbated.

Institutions are typically faced with collecting consent from patients to use their data for research purposes. This incurs a great amount of additional organizational overhead. Also, future collaborations are often not foreseen and in hindsight are not covered by restrictive consent.

Secure multiparty computation (SMPC) is a novel technical approach to this challenge. It allows processing and evaluation of sensitive data and merging of different data pools without the necessity to share the actual patient data with any other institution or party. It therefore solves the conflict between data protection and utilization. SMPC has been applied in various domains in the recent past [1-6]. Moreover, in the medical domain, SMPC has mostly been applied to genomic challenges such as analysis [7], querying [8,9], and computation [10-12]. Furthermore, classification [13] and record-linking problems [14,15] with medical use cases have been addressed. A similar work to ours was performed by Vogelsang et al [16], although it addressed another use case. Their case was the processing of vertically distributed data, where diagnosis data were linked with event records given a common identifier. Our approach focused on the combination of horizontally distributed data, which is needed when performing multicentric studies. Furthermore, in the study by Vogelsang et al [16], they did not perform an in-depth analysis of the influencing factors except for the number of processed items.

In our study, we considered nontrivial algorithms that have widespread use in clinical research and digital health care and aimed to find SMPC versions of them. Survival analysis is particularly prevalent throughout a substantial part of medical literature. On the one hand, survival analysis is essential, for example, to judge the effect of a novel therapy against the current standard or a placebo. On the other hand, individual survival times of patients and their clinical characterizations or traits are highly sensitive data. Here, we turn our attention to a particularly relevant task—the so-called log-rank test [17,18]. It is the most commonly used test to decide if two survival curves (eg, plots of Kaplan-Meier estimators) are significantly different (ie, if there is a significant positive or negative effect of one treatment or trait over another). The realization of a privacy-preserving way of computation is nontrivial because the algorithm requires knowledge of the sorted set of all individual survival times.

Many of today’s practical SMPC realizations are based on circuit representations. The nodes of the circuit represent basic operations, such as addition and multiplication. The circuit itself is then a graph of basic nodes that allow the creation of arbitrary complex functions [19].

Our first objective was to create an SMPC protocol that was able to pool the data from different stakeholders and to process it using the Kaplan-Meier estimator in combination with the log-rank test. We achieved this by first providing a merging algorithm for time-to-event data, which was then used as the basis for the computation of the log-rank test. This meant that any stakeholder’s data did not have to be shared with any other party while enabling the parties to evaluate a common but distributed data set. The implementation was realized with the secret-sharing–based general SMPC framework FRESCO [20], which is written in Java.

Furthermore, as a second objective, we assessed the performance and scaling behavior of the gained realization. In a test setup, we varied the parameters of the participating hosts and the network in between. In a real-world setup, which featured a connection between two research institutions over the internet, we showed practical feasibility of the approach.

## Methods

### Secure Implementation of the Log-Rank Test for the Kaplan-Meier Estimator

Our method for creating secure and privacy-preserving realizations of statistical evaluations of medical data was to rewrite the original algorithms as a protocol for general SMPC. Exemplarily, in this paper, we present a secure implementation of the log-rank test for the Kaplan-Meier estimator.

When applying the Kaplan-Meier estimator to a single data set, the computation can be performed by the data owner while fulfilling the security goal of data confidentiality and keeping individuals’ information private by not sharing it with any third parties. If data are initially distributed among several different stakeholders, the setting becomes more complex: before the log-rank test or other measures can be derived, the data of all sources has to be combined. Merging in itself is an additional step that must be done by some entity and normally requires access to all sets of original data. The data sets consist of nonaggregated survival times of individual patients. This disclosure then constitutes a data protection violation, which normally makes disclosure agreements or other organizational measures necessary.

With SMPC, merging can be realized as a secure protocol between all data owners aiming for confidentiality and data privacy: third-party access to all data becomes superfluous and merging can be performed without the need of sharing original data.

A second privacy problem is that the merged data table can still leak information about individual contributions. This is easily visible in the two-party case: if the merged data are publicly known, both parties can derive the other party’s contributions by simply inverting the merge procedure.

It is therefore necessary to keep the merged data protected from any entity and continue the calculation of the log-rank test without making intermediate results available. We achieved this by not making the result of the merge publicly accessible but directly performing subsequent calculations on the still-protected merged data set.

#### Input Data

Each stakeholder examined several groups of study participants. Without loss of generalization, we assume them to be treatment group A and control group B. For each point in time, *t ∈ T* of the study, the overall sizes of the study groups and the number of events (eg, deaths) during that time in both groups were recorded. We denote them as *risk set_A,t_* and *risk set_B,t_*, as well as *failures_A,t_* and *failures_B,t_*, respectively.

Let *P* be the set of participating stakeholders, each *p* *∈ P* then has a map *entries_p_* of input data, where keys(*entries_p_*) = *T_p_* (ie, all times recorded in the study of *p*) and ∀*t* ∈ *T_p_:*value(*entries_p_*, *t*) = (*risk set_A,t,p_*, *risk set_B,t,p_*, *failures_A,t,p_*, *failures_B,t,p_*). Furthermore, values(*entries_p_*) ≡ *∪_t_*_∈keys(_*_entries_p__*) value(*entries_p_*, *t*).

#### Merging Initially Distributed Data

As outlined above, the first step was to derive a merged data set from the separate data of all stakeholders.

Simply generating *entries*:*∪_p∈P_ entries_p_* was not expedient, since it could not handle duplicate keys in the entries. Instead, a single combined virtual data set had to be created out of the distributed studies by summing up corresponding values of matching keys.

Since the different studies of all stakeholders can contain different points of time, the union set of all times *t* had to be built: keys(*entries*) ≡*∪_p∈P_* keys*(entries_p_*). We obtained this by applying the secure union set algorithm of Blanton and Aguiar [21]. Afterwards, the resulting set was made available in plain for all stakeholders.

Every stakeholder *p* then completed their own *entries_p_* by adding fallback values for all locally missing keys: value(*entries_p_*, *t*) ≡ (*risk set_A,t_prev_ ,p_*, *risk set_B, t_prev_ ,p_*, 0, 0)*∀t* *∈* keys(*entries*) \ keys(*entries_p_*), where *t_prev_* = max(*{t' ∈* keys(*entries*):*t' < t*}), the latest available time. Afterwards, values(*entries_p_*) was turned into a list that was locally sorted by keys(*entries_p_*). This list was provided as input into a simple SMPC protocol, summing up all entries row by row. Since the list features all *t ∈ ∪_p∈P_ T_p_* in the same order, the corresponding entries were summed up correctly. As an intermediate result, we obtained a merged data table as depicted in Table 1. This table was not made available in plain but stayed in a secret shared manner for immediate further processing.

Performing this merge step did not leak any unnecessary information. No values(*entries_p_*) of any party *p* were shared with any other party. Additionally, the mapping *p* → keys(*entries_p_*) for any *p∈ P* remained private in the general case (special cases like n=2 allow the derivation of further information). The only intermediate information that was made available for all parties was the set keys(*entries*). The gained knowledge of party *p* by this intermediate result about the presence of a key *t* only encompassed the following:

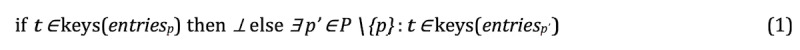



Since the merged table entries themselves are only available in a secret-shared manner, no further information becomes accessible.

**Table 1:** Merged data table containing all times t from all participating stakeholders. If multiple stakeholders provided data for the same t, they were merged by summation.

Time	Risk set		Failures	
	Treatment	Control	Treatment	Control
*t*	∑*_p∈P_ risk set_A,t,p_*	∑*_p∈P_ risk set_B,t,p_*	∑*_p∈P_ failures_A,t,p_*	∑*_p∈P_ failures_B,t,p_*

#### Computation of the Log-Rank Test

The merged data table could then be used to compute the Kaplan-Meier estimator and to perform the log-rank test on it. The secure realization of the computation was structurally identical to its equivalent in plain. The main difference was that the computation was carried out on secret shares of the input data. Consequently, no intermediate values were accessible in plain by the computing parties. It was only the final result (ie, the log-rank value) that was made available in plain to all stakeholders.

### Performance Measurements

Having implemented a secure version of the Kaplan-Meier estimator, we challenged it in both an experimental setting and a real-world setting. In the experimental setting, a range of parameters was varied to investigate the overall performance and scaling of the algorithm. In the real-world setting, distributed computation was performed on actual patient data by the university hospitals of Ludwig-Maximilians-Universität München (Munich) and Charité (Berlin) [22]. Across 500 km of glass fiber cable, we looked for significant variables predetermining the survival time of patients with glioblastoma multiforme, a highly aggressive type of brain tumor.

### Experimental Setting

We analyzed our protocols in two different settings: a testbed and a real-world setup.

#### Testbed

In a controlled testbed setting, we assessed the influence of individual parameters such as the host characteristics or the properties of the network between the cooperating hosts. For the testbed measurement, synthetic data were used.

**Figure 1:**
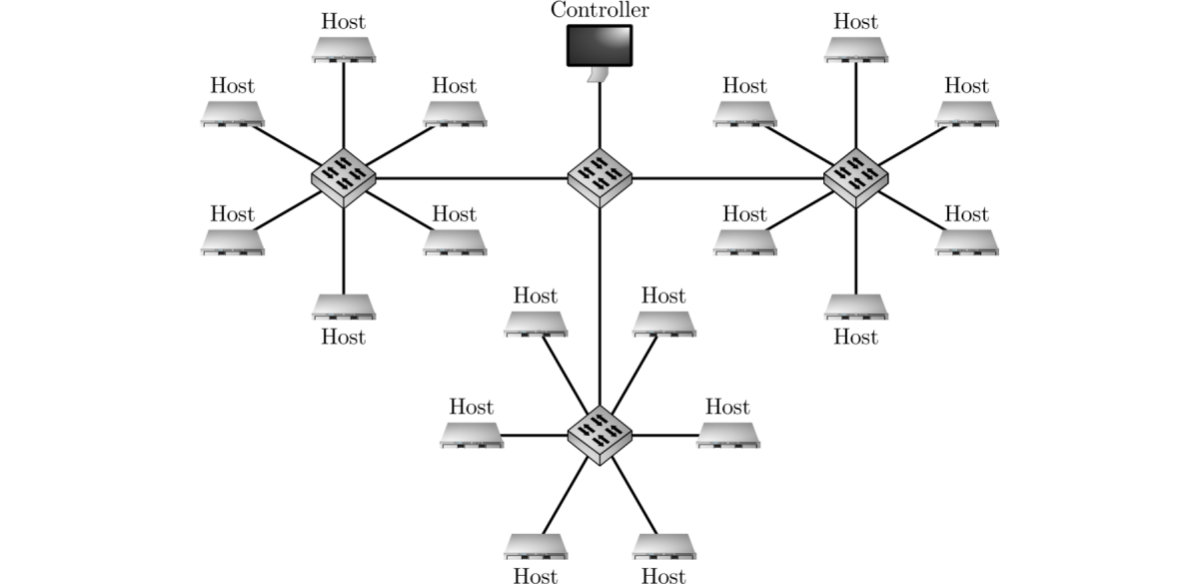
Topology of the testbed setup.

We used homogeneous nonvirtualized bare metal hosts. These were each equipped with an Intel Xeon E3-1265L V2 central processing unit (CPU), having 8 cores at 2.50 GHz and a cache size of 8192 KB. Each host possessed 15,780 MB of RAM and a 1 Gbit networking interface. Six hosts each were connected to a single switch (Figure 1). The operating system was Debian Stretch 9.5 using a kernel of version 4.9.11. We used Java version 11.0.1 2018-10-16 LTS.

#### Real-World Setting

We complemented these evaluations with real-world measurements. In our real-world setup, we cooperated with the University Hospital of Ludwig-Maximilians-Universität München (LMU) and Charité Berlin (CB).

Within the data umbrella of Deutsches Konsortium für Translationale Krebsforschung (DKTK)—of which the Technical University of Munich, University Hospital of Ludwig-Maximilians-Universität München (LMU) and Charité Berlin (CB) are members—the radiation oncology departments of LMU and CB were able to provide glioblastoma survival data [22]. The patient data of LMU and CB contained 96 input entries each.

LMU and CB each provided a server for executing our secure protocols. The server of LMU is equipped with an Intel Xeon Silver 4112 CPU, having 8 cores at 2.60 GHz and a cache size of 8448 KB. It possesses 128,476 MB of RAM and a 1 Gbit networking interface. It provides Debian 9.6 as the operating system using a 4.9 Linux kernel. We used Java version 11.0.2 2018-10-16.

The server of CB uses has an Intel Xeon CPU E5-2695 v3 CPU, with 2 cores at 2.30GHz and a cache size of 35,840 KB. It possesses 3945 MB of RAM and a 10 Gbit networking interface. The host is a VM based on VMWare. It provides Ubuntu 18.04.2 LTS as the operating system using a 4.15 Linux kernel. We used Java 11.0.2 2018-10-16, perf 4.15.18 and tshark 2.6.6. The distance between both servers is approximately 500 km and the protocol was conducted via the open internet.

#### Software

The software under test was the FRESCO framework [20] (version 1.1.2) developed by the nonprofit organization Alexandra Institute. It is a Java framework for SMPC that aims for general application of SMPC on the basis of different mathematical foundations. Each foundation is realized as a protocol suite that comprises basic operations such as addition, multiplication, or Boolean NOT, AND, OR. The FRESCO framework enables users to create protocols for individual computations by combining these protocol primitives into larger sequences.

We employed the protocol suite SPDZ [23,24] in order to develop a secure realization of the Kaplan-Meier estimator and its assessment via the log-rank test. The source code is compiled to a Java application, which is in turn executed by the Java Virtual Machine. At the time of our measurements, only a stable realization of the online phase of SPDZ was available in FRESCO. The offline phase is simulated by a dummy preprocessing. The performance characteristics of the online phase are notwithstanding realistic as if real preprocessing had been performed, and this was confirmed by the authors of FRESCO upon our request [25]. The performance of the offline phase was not considered by our tests.

The source code is available from the authors upon request.

## Results

### Secure Implementation of the Log-Rank Test for the Kaplan-Meier Estimator

The algorithm developed for the secure implementation of the Kaplan-Meier log-rank test is presented in the Multimedia Appendix 1. We made two technical observations: the computation of the mean and the variance of all entries are independent of each other. Their calculation can hence be parallelized to improve execution speed of the algorithm. We denote the regions of possible parallelization in the algorithm with the keyword in parallel. Furthermore, the numerical computation of the variance is prone to overflows. We hence alternated the necessary divisions and multiplications in order to stay within the range of valid values.

In terms of security, our solution was based on the SPDZ implementation of FRESCO and hence inherits its security properties. In particular, this implies computational security against malicious adversaries, which can corrupt up to n-1 out of n parties. At the time of our experiments, FRESCO did not provide a secure implementation of the offline phase SPDZ but only insecure dummy preprocessing. While this does not have any implications for performance, for real application it is vital to replace this with a securely realized preprocessing phase.

To merge data entries from different parties, they have to work on a common set of keys(*entries*). Due to this reason, this intermediate result was made available in plain to all participants. Strictly speaking, this represents an information leakage beyond the final result of the computation.

Under certain circumstances, this can be mitigated: if the keys are discrete integer values and the limits are known in advance, all sets can be prepared to contain all possible keys. As a consequence, consolidation of the key set becomes obsolete and the algorithm can start directly with the summation step of the sorted lists.

### Performance Measurements

In order to assess the following results, we provided two measures of comparison. First, we also implemented the log-rank algorithm insecurely to be carried out on a central server, acting as a trusted third party (TTP); for the measurements, we used the LMU server. Here, a standard Java implementation of the computation has been used. In this case, we only considered the computation itself without network interaction for providing the input data to the server or for sending the result to any recipient.

Second, FRESCO also provides a dummy protocol suite that performs the computation in plain text without execution of secure protocols. The algorithm in question was translated into a circuit representation, but computation was then carried out locally without protocol interaction and corresponding communication. This allowed us to discern the influence of the circuit representation from the actual execution of interactive, synchronized multiparty protocols. We refer to these baselines where appropriate, but do not interpret their performance behavior in greater detail.

#### Correctness of the Computation

The computation was performed as an evaluation of an arithmetic circuit based on the operations of addition and multiplication. All higher-level operations, including division and exponentiation, were also realized upon the aforementioned basic operations. Furthermore, all real values were encoded in fixed-point representation.

This introduced numerical errors into the computation; Table 2 shows the deviation. We could reproduce this behavior with the dummy computation of FRESCO. This led to the conclusion that the deviation was caused by the abovementioned factors and not to the secure computation itself.

Since this effect can in certain cases cause a misleading result, this obstacle has to be further investigated. A possible mitigation is analytical transformation of the corresponding equation in order to yield less division operations. This, however, poses the risk of arithmetic overflows during the computation. They can in turn be addressed on the level of SMPC by increasing the modulus of the secret-sharing scheme. This is a valuable goal for future work.

**Table 2:** Comparison of the results obtained by insecure computation on a trusted third party (TTP) and by secure multiparty computation (SMPC).

Test set	Chi-square (TTP)	*P* value (TTP)	Chi-square (SMPC)	*P* value (SMPC)
Set A	5.242	.02	5.148	.02
Set B	23.250	<.001	20.523	<.001

#### Variation of Input Parameters

**Figure 2:**
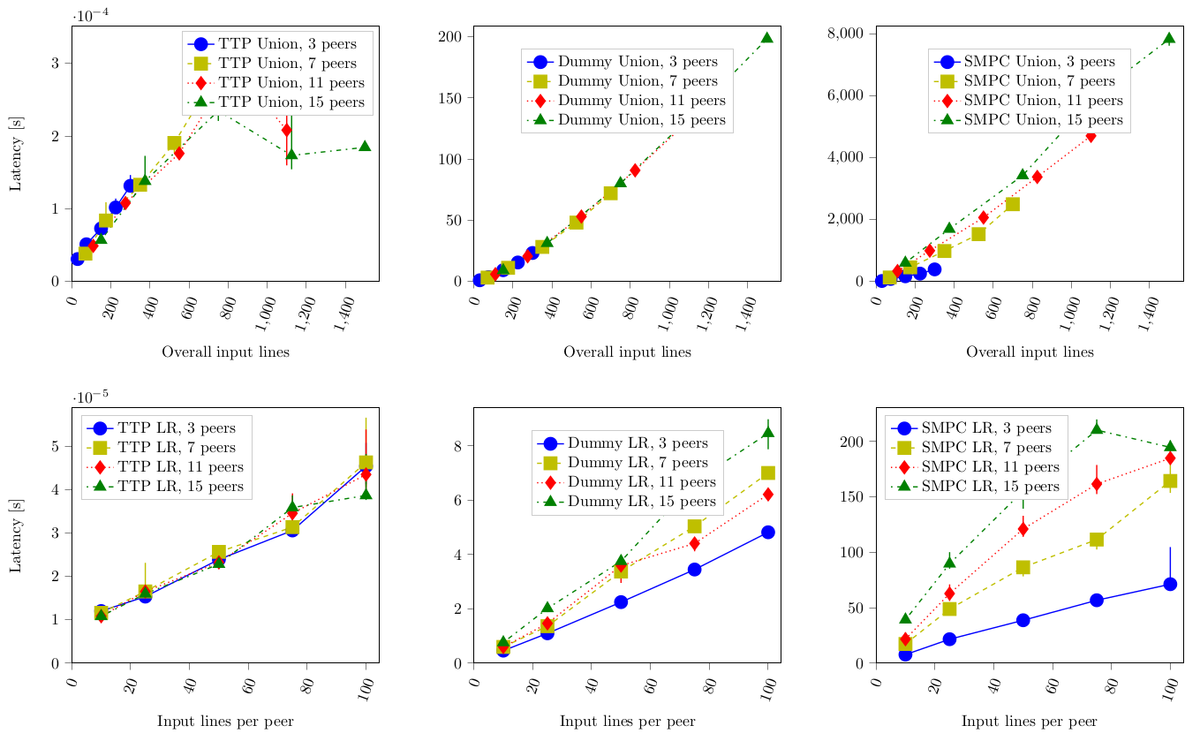
Measurements show that computation times of the corresponding algorithms on a trusted third party (TTP), a dummy implementation, and a real secure multiparty computation (SMPC) vary by orders of magnitudes, depending mainly on the overall number of input lines, with only a subordinate influence of the number of peers, since communication between peers can be parallelized. LR: log-rank.

**Figure 3:**
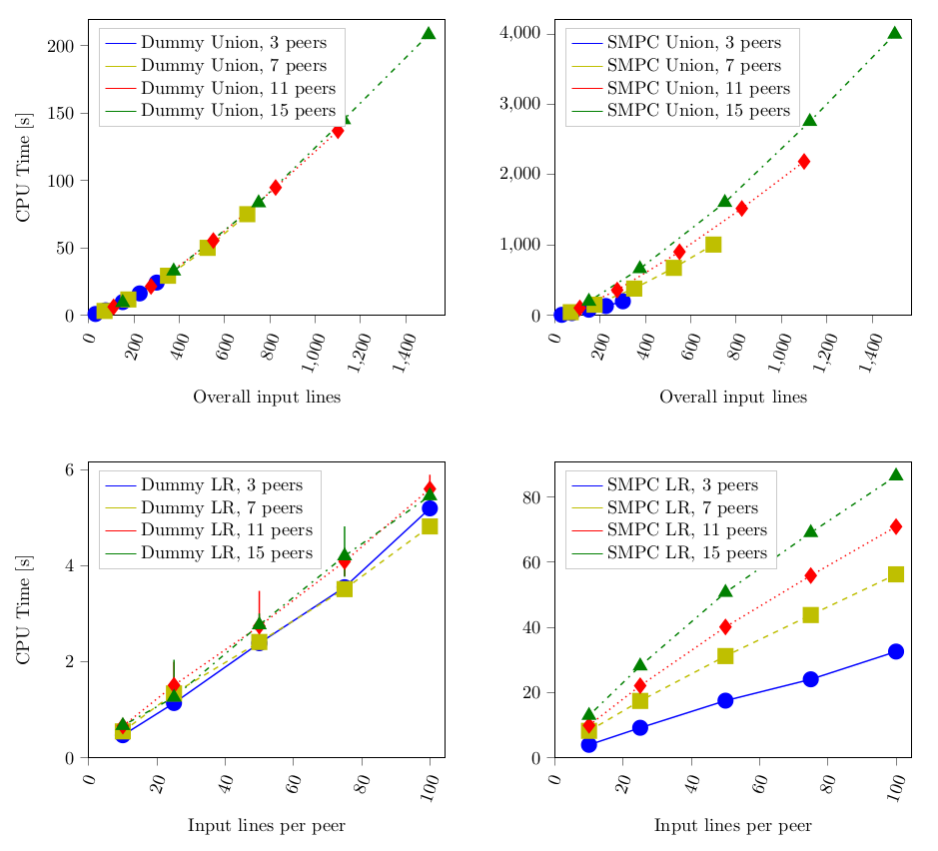
Central processing unit (CPU) time depending on the number of input lines and peers. LR: log-rank.

Comparing all three realizations of the algorithms with respect to execution time, we found that their orders of magnitude differed notably: the TTP variant cost milliseconds, the dummy protocol suite was in the order of seconds, and the secure variant was in the order of minutes (Figure 2). In comparison with Figure 3, we found that the CPU time only constituted between 30% and 50% of the overall execution duration.

Inspection of the log-rank algorithm provided further insights: it showed that the division operation had a much greater impact than any other basic arithmetic operation. The source code of FRESCO states that the Goldschmidt division [26] is used, an approach which iteratively applies multiplications until convergence of the result is reached. For further considerations of the division operation in SMPC and FRESCO in particular, see reference [27]; for further explanation on the application of the Goldschmidt division for SMPC, see reference [28]). When all division operations were replaced by multiplication operations for comparison, the execution time shrank by two orders of magnitude.

Furthermore, it was interesting to see whether the sole number of peers also had an impact on execution duration. For that, we analyzed the dependency of the algorithms on the overall number of input lines. A linear regression on the data yielded a number of insights. For the union algorithm, the following formula held:

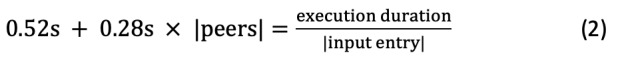



For the log-rank algorithm, we identified a slope of approximately 0. This was in line with our observations in Figure 2: the spread between the different configurations in the latency diagram for the SMPC implementation of the log-rank algorithm can be exclusively explained by the fact that additional peers also add further input entries.

In other words, the time of the union algorithm was mainly influenced by the overall number of input lines (number of peers [n] × number of input lines [m]), notwithstanding whether many peers input few lines or few peers input many lines.

In Figure 3, we can also see that the CPU time proportionally corresponds to the overall execution time. The explanation for different peer configurations was given earlier. The merge step was performed in O(n log n); hence, the lines in Figure 3 initially spread more and converge against the same slope. We can also see that the CPU is moderately more utilized when having more participating peers. The reason are the steps necessary to manage and perform communication with other peers (notwithstanding the communication delay itself).

**Figure 4:**
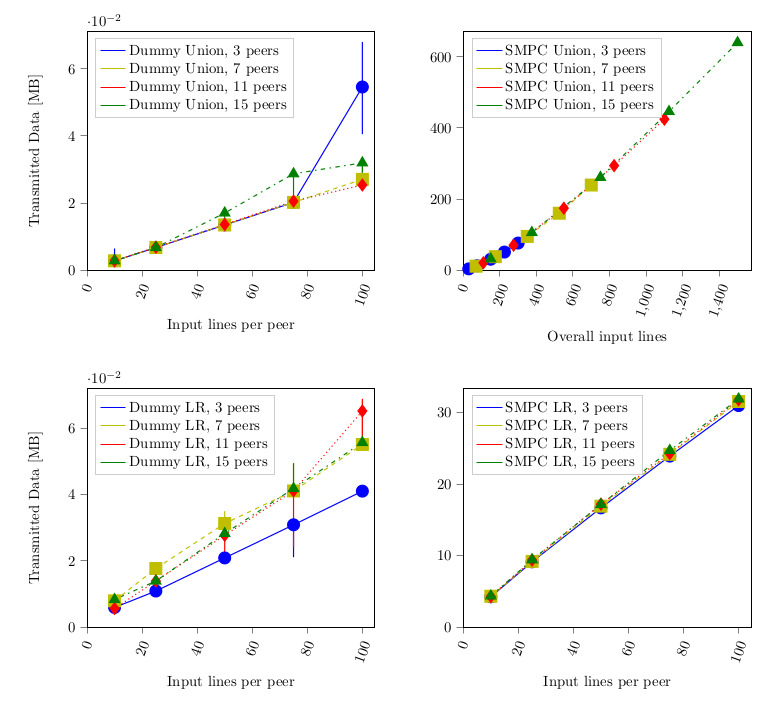
Transmitted data depending on the number of input lines and peers. The graph depicts the number of megabytes transferred between a single pair of hosts in the network. In the secure multiparty computation (SMPC) case, they nearly perfectly correlate with the amount of protocol invocations. LR: log-rank.

In Figure 4, we depict the transmitted data between a single pair of hosts. Compared with the dummy protocol, the SMPC implementation again differs by orders of magnitude. The reason is that computation in plain (as given in the dummy implementation) is able to do some computations (especially the basic multiplication) without any communication, while for SMPC exchange, communication is necessary every time such an operation takes place. We stress that the data shown in the graphs reflect the communication of a single pair. There were n^2^ such pairs during each computation, and thus the overall amount of transmitted data over the network increased accordingly. We could verify by inspection that the amount of transmitted data was equally sized for every pair. Furthermore, the majority of packets had a size of approximately 200 bytes, independent of the number of peers or input lines.

We already elaborated that the log-rank algorithm was made independent of the number of peers by the initial data merging step. This was also confirmed by these measurements, which show that the amount of transmitted data did not depend on the number of participating peers.

#### Variation of Resource Parameters

After we analyzed the basic behavior when scaling environment parameters such as the number of input lines and the number of peers, we then addressed the technical parameters of the setup. This encompassed the network latency, the transmission rate, and the cores and frequency of the CPUs used.

#### Network Latency

**Figure 5:**
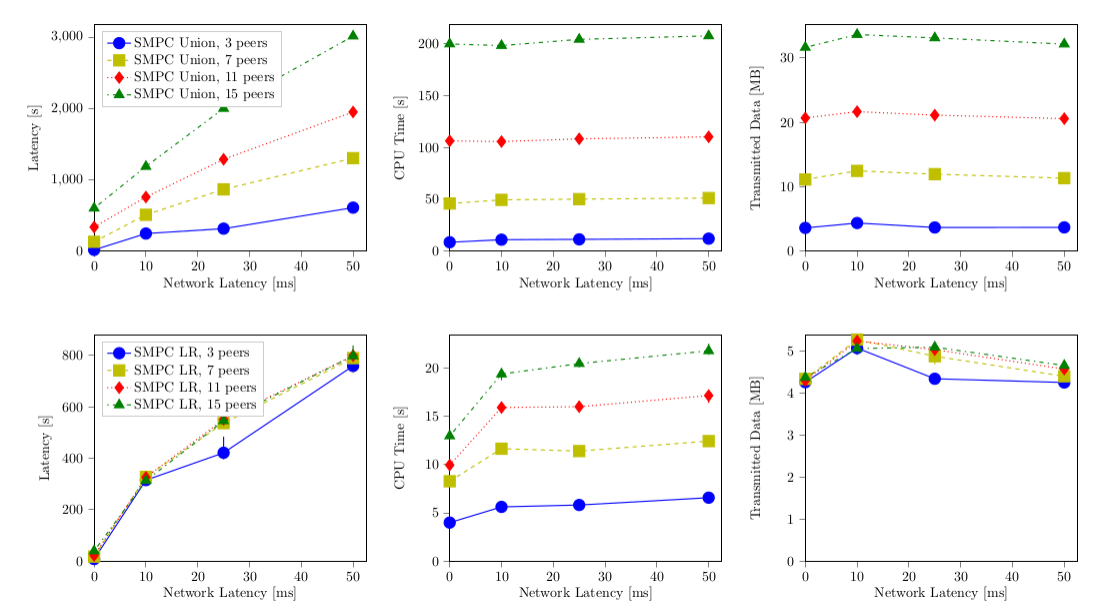
Influences of network latency manipulation. The upper row shows the union algorithm, the lower row shows the log-rank (LR) algorithm. It is clear that the network latency influenced the overall execution time while changing neither the central processing unit (CPU) time nor the number of packets transmitted. SMPC: secure multiparty computation.

Figure 5 demonstrates the influence of increased packet delay on the computation. We already showed in Figure 4 that more data were transmitted during the union algorithm than during the log-rank algorithm. Furthermore, we found that for the majority the packet size stayed roughly the same notwithstanding the variations of the parameters. Hence, with a rather constant number of packets, it was expected that packet delay influenced the union algorithm correspondingly stronger than the log-rank computation. The slight variations in the amount of transmitted data over the different network latencies can be explained by variation in the average packet size. With a latency of 10 ms, the packet size was roughly 80 to 100 bytes smaller. To transport the same amount of payload, more packets were needed. This yielded an increase of transferred headers, which in turn caused an increase in the total amount of data transmitted.

The CPU time was not influenced by the packet delay; it stayed completely constant for the union algorithm and only varied slightly for the log-rank computation.

#### Transmission Rate

Transmission rate only inhibited the computation if it was under 10 Mbit/s. More specific inspection of the network traces showed that our use case continuously used approximately 2 Mbit/s with short but high peaks during the log-rank computation. The union algorithm was characterized by a rather consistent stream of packets of the named rate.

#### CPU Frequency and Number of Cores

We varied the number of cores between 1 and 8, and the frequency could be adjusted from 100% (ie, 2.5 GHz) down to 50% (ie, 1.25 GHz). A lower value was not possible with our test machines. Notwithstanding a varying number of peers, the changes did not yield any significant influence on the execution duration of the algorithms. We conclude that the CPU did not constitute the bottleneck in our test setting.

### Real-World Experiments

In the real-world experiments, we executed our protocol between two servers from different research institutions. The round-trip time is approximately 9 ms from LMU and 21 ms from CB. The transmission speed (approximated using netcat) is approximately 800 Mbps from LMU to CB and 100 Mbps from CB to LMU.

Due to the predetermined setup, we did not vary most of the parameters as we did in the testbed. We only changed the number of input entries per peer from 10 to 96, which produced computation durations between 80 s and 577 s for the union algorithm and 182 s to 652 s for the log-rank algorithm. These numbers were highly influenced by the network latency between both hosts. This outweighed the small number of participants. This interpretation was also supported by the small percentage of CPU time. For the union algorithm, the CPU time ranged from 3% to 9%; for the log-rank algorithm, it was even smaller, ranging from approximately 2% to 5%. The overall execution time for both algorithms lay approximately in the same range. Although this result was in line with our previous observations, the effect became more clear in this setting. The reason can be found in the different type of data used. With our synthetic data, each of the n peers had m input lines. The set of keys was identical for each peer. Therefore, the merge step (which occurred at the beginning of our log-rank implementation) reduced the overall number of lines by factor n, and the remaining steps of the log-rank algorithm always had to compute with m lines only. In contrast, the real data used here had only a negligible amount of identical keys. This meant that the log-rank algorithm always had to process roughly n×m lines. This increased the time taken by the log-rank algorithm. On the other hand, only having two peers reduced the interval in which we tested the union algorithm. For these two reasons, the execution times of both algorithms moved into the same range.

The question arose of whether the measurement results were in line with our testbed results in terms of absolute numbers. For that, we did not use the dimension of wall-clock time since we already knew that it would not match because of the differences in the network latency of the used connections. Instead, the number of protocol invocations and the amount of transferred megabytes were expedient characteristics for comparison because of their independence from time.

In order to obtain a valid comparison, we had to rescale the results. For the union algorithm, we always considered the overall number of input lines by multiplying the input per peer with the number of peers. For the log-rank algorithm, we made a case differentiation. From the testbed measurements, we chose the results by the number m of inputs per peer (since the merge step reduced all n×m inputs to effectively m lines), and from the real-world measurements, we directly considered the product n×m since the merge step did not reduce the input here.

Tables 3 and 4 list the chosen results from the testbed and the real-world setting. They represent the median values of the corresponding measurements. We can see that the real-world results fall between the results from the testbed within an expected range of precision. This is true for both the union algorithm and the log-rank algorithm. An overview of the most important results from the real-world measurements is given in Multimedia Appendix 2.

**Table 3:** Comparison of the testbed and real-world measurement results for the union algorithm (median values).

Setting	Input lines^a^	Protocol invocations	Megabytes
Testbed	75	4954207	12.279469
Real-world	100	7382400	16.681843
Testbed	150	13121941	30.64034
Real-world	150	13121996	29.262298
Testbed	175	16236258	37.808268
Real-world	200	18163340	39.410625
Testbed	225	22505986	50.786938

^a^Input lines refer to the overall number from the whole set of participants.

**Table 4:** Comparison of the testbed and real-world measurement results for the log-rank algorithm (median values).

Setting	Input lines^a^	Protocol invocations	Megabytes
Testbed	50	2659443	16.929702
Real-world	50	2497182	14.860479
Testbed	100	5302094	31.7901285
Real-world	100	5033945	28.212929

^a^Input lines refer to the number of lines the log-rank algorithm had to process after the merge step.

## Discussion

### Principal Findings

We have presented a secure implementation of the log-rank test for the Kaplan-Meier estimator. Our measurements showed that the most influential inherent factor was the number of certain mathematical operations, such as division. The most influential environmental factor was network latency. In a real-word experiment, we successfully demonstrated distributed computing between two university hospitals on actual patient data of glioblastoma survival.

#### Influential Inherent Factors

In general, the time heavily depends on the complexity of the algorithm and the selection of operations used. If the set of operations is well supported, the execution time can be in the realm of milliseconds. If real numbers, division, or comparison operations are used, execution time quickly exceeds seconds to become minutes. Also, depending on the complexity of the computation, each pair of peers exchanges at least some megabytes of traffic. This can also quickly increment to hundreds of megabytes (eg, when sorting).

We identified that the division operation is orders of magnitude more costly than any other basic arithmetic operation. This is the single most influential internal performance factor in the log-rank algorithm.

#### Influential Environmental Factors

Regarding influential environmental factors, we found that network latency has the strongest impact and produces the typical bottleneck. The reason is that the network communication consists of a large number of small-sized packets. The transmission rate does not constitute a bottleneck if at least 2 Mbit/s are guaranteed. We could estimate this lower bound by inspection. Manipulation of the CPU did not yield any changes. We assume that the CPU would have to be constrained to a small fraction of its normal power to achieve any effects. This was not possible in our setups.

As a consequence of the high influence of the network, it is difficult to improve performance characteristics by hardware changes. The most obvious approach of improving the participating hosts did not address the bottleneck. We found that CPU time constituted approximately 30% to 50% of the computation. Here, only moderate improvements by an increased CPU frequency can be expected. On the contrary, every reduction of network latency would be worthwhile.

### Conclusions

Medical studies provide an essential benefit for society. Having a large basis of test subjects improves the validity and robustness of the obtained results. In so-called multicentric studies, this is exploited by letting several institutions carry out the same study with different participants. The gathered data are then merged. However, data protection regulations make the combination of data from different sources more difficult in certain cases and require a notable organizational overhead to fulfill the protection requirements.

SMPC is a promising solution that allows aggregation of study data without actually sharing it between participating centers. In this paper, we investigated how SMPC can be applied in this domain.

The specific contribution of the presented implementation to the field is the ability to derive a relevant quantity, the log-rank *P* value, directly from data sets distributed between several medical institutions without the need to pool the data at a central location. For example, in the recent work by Vogelsang et al [16], the Kaplan-Meier estimator itself was aggregated from distributed input data. Of course, this is a perfectly valid approach if the estimator itself is considered the desired output of the calculation. The log-rank test could then be classically performed on this aggregated estimator. However, if one is only interested in the *P* value (and probably a whole set of *P* values from different cross sections), then our implementation leaks a lot less information as the implicit Kaplan-Meier estimator remains secret. Given general SMPC frameworks like FRESCO, realizations of computations for medical analysis are achievable with acceptable effort.

Having obtained a secure implementation, we conducted thorough performance measurements of this solution. We investigated the impact of peers and input data on the duration, CPU time, and data transmission. Furthermore, we evaluated the impact of selected network and host parameters on the computation time and resources.

We conducted the aforementioned measurements in a synthetic testbed with homogeneous hosts that were connected via an intranet. To complement our insights and gain further knowledge about SMPC performance in real settings, we also performed measurements in a real-world setting with heterogeneous hardware over the internet. For that, two medical institutions provided locally distributed servers where our solution was carried out, and we were able to confirm our results from the testbed.

Our results show that realization of secure computation for medical research is possible with the current state of SMPC. Furthermore, performance measurements indicate that practical application is also already possible.

In the future, more advanced methods such as the Cox proportional hazard model should also be written as SMPC algorithms. Furthermore, the identified obstacles should be addressed: the loss of accuracy compared with plain text calculations should be further reduced or eliminated. Similarly, ways should be found to avoid the intermediate results between the merging step and the arithmetic calculation of the log-rank test result.

However, the main challenges to be addressed going forward may be those of a less technical nature. Over time, many more practically relevant algorithms will be translated into secure variants. After all, the universality of SMPC guarantees solutions for any problem, at least in principle. What will be more relevant to the practical application, however, will be the standardization of protocols, interfaces, and libraries. Just as important will be the inclusion of data protection officers and other stakeholders in the design of an overarching ecosystem for secure distributed computing, including organizational, operational, and conceptual designs. We hope that our real-life demonstration of technical feasibility contributes to the motivation for further research and activities in this relevant and developing field.

## Appendix

Multimedia Appendix 1 Secure Kaplan-Meier estimation with log-rank test.

Multimedia Appendix 2 The results of the real-world measurement of the Kaplan-Meier estimator and its log-rank test evaluation.

## Abbreviations

CPU: central processing unit
SMPC: secure multiparty computation
TTP: trusted third party
