# Supplementary material for: A Privacy-Preserving Log-Rank Test for the Kaplan-Meier Estimator With Secure Multiparty Computation: Algorithm Development and Validation
Source: JMIR Med Inform. 2021 Jan 18;9(1):e22158. doi: 10.2196/22158 (PMC7850908; doi:10.2196/22158)
Supplement: Multimedia Appendix 1 [file medinform_v9i1e22158_app1.docx]

## Multimedia Appendix I

Listing 1: Secure Kaplan–Meier Estimation with Log-Rank Test

1 compute Expected ( entry ):

2 compute in sequence :

3 failures = add ( entry . failure_ treatment , entry . failure_ control )

4 risk_ set = add ( entry . riskset_ treatment , entry . riskset_ control )

5 e = div ( mult ( entry . riskset_ treatment , failures ), risk_ set )

6 return e

7

8 compute Variance ( entry ):

9 compute in sequence :

10 failures = add ( entry . failure_ treatment , entry . failure_ control )

11 risk_ set = add ( entry . riskset_ treatment , entry . riskset_ control )

12 v = div ( mult ( div ( mult ( div ( mult ( failures , sub ( risk_set , failures )), risk_ set ), entry . riskset_ treatment ), risk_ set ), entry . riskset_ control ), sub ( risk_set , 1))

13 return v

14

15 log_ rank ( entries ):

16 compute in sequence :

17 expected = []

18 variances = []

19 ft Entries = []

20 for entry in entries do in parallel :

21 expected << compute Expected ( entry )

22 variances << compute Variance ( entry )

23 ft Entries << entry . failure_ treatment

24

25 compute in parallel :

26 expected_ failure = sum ( expected )

27 failures_ treatment = sum ( ft Entries )

28 variance = sum ( variances )

29

30 diff = sub ( expected_ failure , failures_ treatment )

31 log Rank = div ( mult ( diff , diff ), variance )

32 return open ( log Rank )
