# Supplementary material for: A Privacy-Preserving Log-Rank Test for the Kaplan-Meier Estimator With Secure Multiparty Computation: Algorithm Development and Validation
Source: JMIR Med Inform. 2021 Jan 18;9(1):e22158. doi: 10.2196/22158 (PMC7850908; doi:10.2196/22158)
Supplement: Multimedia Appendix 2 [file medinform_v9i1e22158_app2.docx]

# Multimedia Appendix 2

|  |  |  | Protocol invocations | Protocol batches | Latency [s] | CPU Time [s] | Transmitted Data [MBytes] |
| --- | --- | --- | --- | --- | --- | --- | --- |
| algorithm | algo_part | input_entries | |  |  |  |  |
| km_dummy | logrank | 10 | 1618839 | 44366 | 0.4540 | 0.460* | 0.007821 |
|  |  | 25 | 7461523 | 44396 | 2.3300 | 2.050 | 0.030459 |
|  |  | 50 | 15040469 | 44431 | 5.4220 | 4.405 | 0.060291 |
|  |  | 75 | 22936687 | 44488 | 9.0850 | 7.175 | 0.091311 |
|  |  | 100 | 29569694 | 44549 | 11.9015 | 9.620 | 0.104156 |
|  | union | 10 | 1172362 | 58517 | 0.6655 | 0.710 | 0.001939 |
|  |  | 25 | 4731855 | 81844 | 2.0360 | 2.115 | 0.004051 |
|  |  | 50 | 12850823 | 109151 | 5.0965 | 5.225 | 0.007549 |
|  |  | 75 | 22833876 | 140383 | 8.9675 | 9.135 | 0.010849 |
|  |  | 100 | 31601773 | 140569 | 12.5840 | 12.745 | 0.013819 |
| km_smc | logrank | 10 | 1070176 | 14024 | 182.1695 | 4.230 | 6.952198 |
|  |  | 25 | 2497182 | 14043 | 289.9070 | 8.355 | 14.860479 |
|  |  | 50 | 5033945 | 14067 | 420.4515 | 15.535 | 28.212929 |
|  |  | 75 | 7676495 | 14096 | 529.6670 | 22.900 | 41.959586 |
|  |  | 100 | 9896507 | 14119 | 652.0690 | 30.070 | 53.848914 |
|  | union | 10 | 671681 | 37297 | 80.2470 | 2.625 | 2.143541 |
|  |  | 25 | 2715944 | 52157 | 165.5565 | 8.230 | 6.680932 |
|  |  | 50 | 7382400 | 69532 | 308.7455 | 21.425 | 16.681843 |
|  |  | 75 | 13121996 | 89429 | 499.7760 | 38.105 | 29.262298 |
|  |  | 100 | 18163340 | 89500 | 576.9665 | 52.935 | 39.410625 |

Table 5:The results of the real-world measurement of the Kaplan–Meier estimator and its log-rank test evaluation. We always show the median value of 10 runs per configuration.

* The CPU measurements are invoked at a higher level of the program code than the time measurements. Therefore, it takes a slightly higher but constant overhead
